# Analysis of Five Biogenic Amines in Foods on the Chinese Market and Estimation of Acute Histamine Exposure from Fermented Foods in the Chinese Population

**DOI:** 10.3390/foods14142550

**Published:** 2025-07-21

**Authors:** Pei Cao, Mengmeng Gao, Dongmei Huang, Xiaomin Xu, Zhujun Liu, Qing Liu, Yang Lu, Feng Pan, Zhaoxin Li, Jinfang Sun, Lei Zhang, Pingping Zhou

**Affiliations:** 1 China National Center for Food Safety Risk Assessment, Beijing 100022, China; 2 School of Basic Medical Sciences, North China University of Science and Technology, Tangshan 063509, China; 3 East China Sea Fisheries Research Institute, Chinese Academy of Fishery Sciences, Shanghai 200090, China; 4 Zhe Jiang Provincial Center for Disease Control and Prevention, Hangzhou 310051, China; 5 School of Public Health, Southeast University, Nanjing 210009, China; 6 He Bei Provincial Center for Disease Control and Prevention, Shijiazhuang 050021, China; 7 Yellow Sea Fisheries Research Institute, Chinese Academy of Fishery Sciences, Qingdao 266071, China

**Keywords:** biogenic amines, acute exposure assessment, probability assessment, histamine, acute health risks

## Abstract

Biogenic amines (BAs) are frequently detected in seafood products, wines, and fermented foods, and they pose potential risks to human health. The current study analyzed the concentrations of five common BAs in seafood, fermented food, and complementary food for infants and children (fish sausage, canned complementary food for infants containing fish and shrimp ingredients, and fish floss) in China and estimated the acute health risks of histamine (HIS) from fermented foods in Chinese consumers. Among all the samples analyzed, HIS exhibited the highest detection rate (51.9%), followed by PUT (50.1%), and the detection rate of TRY (12.5%) was the lowest. The total average concentration of the five BAs across major food categories revealed that fermented bean curd had the highest total concentration of BAs (816.8 mg/kg), followed by shrimp (383.2 mg/kg) and cheese (328.0 mg/kg). In contrast, samples of complementary food for infants and children contained the lowest concentrations of BAs; the total average concentration of the five BAs was 12.0 mg/kg. The point assessment results showed that acute dietary exposure to HIS was highest from cheese (76.2 mg/d), followed by fermented bean products (74.5 mg/d). Furthermore, the probability assessment indicated that the probability of acute health risks from exposure to HIS was 0.44% for fermented bean product consumers and 0.014% for cheese consumers, respectively. Thus, for the general consumer, the probability of acute health risks caused by HIS in seafood and fermented foods is low. However, individuals with high consumption of cheese and fermented bean products may need to be concerned.

## 1. Introduction

Biogenic amines (BAs), a class of biologically active low-molecular-weight nitrogenous organic compounds, are primarily synthesized through cellular metabolic processes involving either the decarboxylation of amino acids or the amination and transamination of aldehydes and ketones [1,2]. Studies have demonstrated that BAs, including histamine (HIS), tyramine (TYR), putrescine (PUT), and cadaverine (CAD), are frequently detected in various food products, especially in seafood products, wines, and fermented foods [3,4,5,6,7]. While BAs are essential components of living cells and play critical physiological roles in processes such as protein synthesis, cell growth and differentiation, and intestinal immunity, the consumption of foods containing high levels of BAs may potentially lead to adverse health effects [8,9]. HIS is recognized as the causative agent in a number of foodborne intoxication outbreaks and exhibits the highest toxicity among Bas [10]. Acute HIS poisoning affects the vascular system and smooth muscles, presenting clinically as headache, bronchospasm, tachycardia, hypotension, urticaria, and asthma [8,11]. A certain amount of TYR and TRY induce vasoconstriction, potentially leading to hypertension, accompanied by headache and vomiting [10]. In contrast, PUT and CAD exhibit relatively low toxicity, primarily causing hypotension and bradycardia [10].

Due to the hazards of BAs, some countries have established regulatory limits for BAs in food products, with a primary focus on HIS due to its health risks. For example, the U.S. Food and Drug Administration (FDA) requires that the level of HIS in fish and fish products not exceed 50 mg/kg [12]. Similarly, the European Union (EU) has implemented specific HIS limits for high-HIS fish products (they should not exceed 100 mg/kg or 100–200 mg/kg) and high-HIS fish products treated with enzymes in saltwater (they should not exceed 200 mg/kg or 200–400 mg/kg) [10,13]. In addition to aquatic products, several countries have also established HIS limits in wine, such as 2 mg/kg in Germany, 3.5 mg/kg in the Netherlands, and 5 mg/kg in Finland [14,15]. In China, regulatory measures have been implemented to control HIS levels in specific food categories. For example, the Chinese government has established HIS limits of 400 mg/kg for high-HIS fish species (such as mackerel, tuna, and sardines) and 200 mg/kg for other fish products (GB 2733-2015) [16,17]. Additionally, canned products derived from mackerel, caranx, and sardines are subject to a higher HIS limit of 1000 mg/kg, as specified in GB 7098-2015 [18]. However, it is noteworthy that China has not yet established regulatory limits for BAs in other food categories.

In 2011, the European Food Safety Authority (EFSA) conducted a comprehensive qualitative risk assessment of BAs (HIS, TYR, PUT, CAD, PHE, and TRY) in fermented foods (such as alcoholic beverages, fish and fish products, meat products, dairy products, fish sauce, and fermented vegetables). Based on the No Observed Adverse Effect Level (NOAEL) of 50 mg for HIS in both healthy volunteers and sensitive individuals, an Acute Reference Dose (ARfD) for HIS in healthy adults was established [10]. The assessment indicated that healthy adults consuming less than 50 mg of HIS and 600 mg of TYR per meal were at a lower risk of experiencing adverse health effects [10]. Subsequently, in 2012, the Food and Agriculture Organization of the United Nations/World Health Organization (FAO/WHO) and the Joint Expert Committee on Food Additives (JECFA) published a report on the risk assessment of HIS in fish and fish products [19]. The report revealed that HIS concentrations exceeding 200 mg/kg in fish and fishery products may pose significant health risks to consumers [19]. In China, numerous cases of HIS poisoning have been reported over the past decade, primarily associated with the consumption of scombroid fish such as blue mackerel and mackerel [16,20,21]. Furthermore, studies have shown that some fermented foods such as Chinese fermented bean products (including fermented bean curd, fermented soya bean), cheese, and fermented wine in China are contaminated with BAs, which may pose potential health risks for consumers [22,23,24]. However, limited studies have been reported in China regarding the risk assessment of BAs in foods.

The aim of this study was to analyze the concentrations of five BAs (HIS, TYR, PUT, CAD, and TRY) in seafood, fermented foods, and complementary food for infants and children in China. Due to only HIS having a temporary ARfD established by EFSA, this study assesses the acute health risks of HIS exposure from fermented foods in Chinese consumers. This study will provide a scientific assessment basis for the risk management of five common BAs in foods in China.

## 2. Materials and Methods

### 2.1. Sample Collection and Preparation

A total of 1200 samples were collected, including 485 from seafood, 112 from seafood products, 81 from cheese, 96 from fermented alcoholic beverages, 152 from fermented vegetable products, 76 from fermented meat products, 75 from fermented bean products, 38 from fish sauce, and 85 from complementary foods for infants and young children. The category of “seafood” was divided into the subcategories of “marine fish”, “shrimp”, and “mollusks or cephalopods”. “Seafood products” was divided into “dried fish”, “dried scallops”, “shrimp paste”, and “canned fish”. “Fermented alcoholic beverages” was divided into “huangjiu”, “beer”, and “wine”. “Fermented bean products” was divided into “fermented black beans”, “fermented bean curd”, and “other fermented bean products”. “Complementary foods for infants and young children” was divided into “fish sausage”, “canned complementary foods for infants and young children” (containing fish and shrimp ingredients), “fish floss”, and “other canned complementary foods for infants and young children”. Furthermore, other fermented bean product samples included stinky tofu, moldy tofu, and soybean paste. The cheeses studied included goat’s milk cheese and cow’s milk cheese. The wines studied included red wine and white wine. The samples were selected from the most popular brands in China and were obtained from major markets and online shopping platforms in Fujian (100), Henan (100), Hubei (108), Hunan (106), Sichuan (107), Zhejiang (106), Hebei (255), Shanghai (150), and Qindao (150). Each sample was individually collected in a sealed bag with ice and transported to the laboratories of the Center for Disease Control and Prevention in the above-mentioned provinces/cities immediately. The edible portions of the samples were homogenized by stirring and blending. Solid samples were stored at temperatures below −18 °C, while liquid samples were stored at temperatures below 4 °C.

A total of 2.0 g of the samples was placed into a 50 mL centrifuge tube. Subsequently, 20 mL of 5% HClO was added to the tube, and the mixture was vortex-mixed for 5 min, followed by ultrasonic cleaning for 20 min. Using a refrigerated centrifuge, the mixture was centrifuged at 5000 rpm for 10 min. The remaining solid was re-extracted with 20 mL of 5% HClO. To both extracts, 400 g/L of NaOH was added, and the pH was adjusted to 2–3. Then, the solution was combined with 0.5% HClO in a 50 mL volumetric flask. A total of 2.0 mL of the solution was transferred to a centrifuge tube, and 2.0 mL of 0.5% HClO and 5.0 mL of n-hexane were added. After purification with n-hexane, the cleaned solution was diluted to 1.0 mL with 0.5% HClO. Prior to HPLC-MS/MS analysis, the solution was filtered through 0.22 μm polytetrafluoroethylene (PTFE) syringe filters (Tianjin, China) to remove particulates prior to injection [25].

### 2.2. Chemicals and Reagents

Histamine dihydrochloride (C_5_H_9_N_3_·2HCl, CAS No. 56-92-8, purity > 99%), tyramine hydrochloride (C_8_H_11_NO·HCl, CAS No. 60-19-5, purity > 98%), putrescine dihydrochloride (C_4_H_12_N_2_·2HCl, CAS No. 333-93-7, purity > 99%), cadaverine (C_5_H_14_N_2_, CAS No. 462-94-2, purity > 98%), and tryptamine (C_10_H_12_N_2_, CAS No. 61-54-1, purity > 98%) were purchased from Dr. Ehrenstorfer GmbH (Augsburg, Germany). All these standards were stored at −20 °C and kept away from light. Perchloric acid (HClO, 70–72%) and sodium hydroxide (NaOH) were obtained from Sinopharm Group Chemical Reagent Co., Ltd. (Shanghai, China). Ammonium formate (HPLC-grade) was purchased from Honeywell (Morris Plains, NJ, USA). Chromatographic grade formic acid, acetonitrile, and n-hexane were obtained from Aladdin Reagent Co., Ltd. (Shanghai, China). Deionized water was prepared using a Milli-Q Ultra Pure Water System (Millipore, Billerica, MA, USA).

### 2.3. Standard Solution Preparation

Stock standard solutions of HIS, TRY, CAD, PUT, and TYR at a concentration of 5.0 g/L were prepared by dissolving the respective compounds in a 10 mL volumetric flask with deionized water. A mixed standard intermediate solution (200 mg/L) containing CAD, PUT, and TYR was prepared by adding 0.4 mL of the stock standard solutions of CAD, PUT, and TYR, respectively. Another mixed standard intermediate solution (20 mg/L) containing HIS and TRY was prepared by adding 0.2 mL of the stock standard solutions of HIS and TRY, respectively. The mixed working standard solution of the five BAs was prepared by combining 0.1 mL of the mixed standard intermediate solution of CAD, PUT, and TYR with 0.1 mL of the mixed standard intermediate solution of HIS and TRY. To prepare the standard series solutions, 0.05 mL, 0.125 mL, 0.25 mL, 0.5 mL, 1.0 mL, 2.5 mL, and 5.0 mL of the mixed working standard solution of the five BAs were transferred into a 10 mL volumetric flask and diluted with a 0.5% perchloric acid–acetonitrile solution [25]. All prepared solutions were stored at 4 °C in the dark to ensure stability.

### 2.4. LC-MS Analysis

A 5500 QTRAP triple quadrupole LC-MS (AB SCIEX, Frawanda, MA, USA), coupled with an LC-30 AD HPLC system (Shimadzu, Kyoto, Japan) and an ACQUITY HSS T3 column (2.1 mm × 100 mm, 1.8 µm, Watts, Milford, MA, USA), was employed for the analysis [25]. Mobile phase A was an aqueous solution containing 10.0 mmol/L ammonium formate and 0.5% formic acid, while mobile phase B was an acetonitrile solution containing 10 mmol/L ammonium formate and 0.5% formic acid. The gradient elution program is presented in Table 1. The injection volume of the mobile phase was set at 2.0 µL, the flow rate was maintained at 0.3 mL/min, and the column temperature was kept at 35 °C. The mobile phase was eluted at a flow rate of 0.3 mL/min. The quantitative ion chromatograms of standard solutions for CAD, PUT, TYR, HIS, and TRY are presented in Figure S1.

Mass spectrometric analysis was performed in multiple reaction monitoring (MRM) mode under the following optimized conditions: The ionization mode was set to positive electrospray ionization (ESI+). The spray voltage was maintained at 5500 V, and the temperature of the ion transfer capillary was set to 550 °C. The curtain gas pressure (CUR) was 40 psi, while the nebulizing gas pressure (GS1) and auxiliary heating gas pressure (GS2) were both set to 55 psi. The collision gas (CAD) pressure was maintained at 5 psi. The specific MRM transitions, including parent ions, daughter ions, and corresponding collision energies, are detailed in Table 2.

### 2.5. Analytical Validation

To ensure the reliability and accuracy of the analytical results, internal quality controls were employed across all participating laboratories. The method precision (intra-day repeatability, RSD_r_) and accuracy (percentage recoveries) were estimated through recovery experiments in each laboratory. The results demonstrated that the percentage recoveries of HIS, TYR, CAD, PUT, and TRY ranged from 75.5% to 106.0%, 78.7% to 113.0%, 76.8% to 108.0%, 74.1% to 102.0%, and 71.7% to 98.0%, respectively. The corresponding RSD_r_ varied from 1.11% to 6.58%, 0.95% to 9.14%, 1.5% to 8.35%, 1.48% to7.74%, and 1.32% to 5.75%, respectively. These results indicate that the precision and accuracy of the method satisfy the predefined quality control criteria for this study, thereby ensuring the robustness and reliability of the analytical approach.

### 2.6. Acute Dietary Exposure Assessment of Five BAs from Seafood and Fermented Foods Among Chinese Consumers

#### 2.6.1. Consumption Data of Seafood and Fermented Foods in Chinese Consumers

The seafood and fermented food consumption data used in this study were obtained from the China National Food Consumption survey, which was conducted between 2018 and 2020. This survey employed a multistage random cluster sampling and was implemented across 18 provinces in China, including Beijing, Tianjin, Hebei, Neimeng Gu, Liaoning, Jilin, Heilongjiang, Jiangsu, Zhejiang, Fujian, Jiangxi, Shandong, Henan, Hubei, Hunan, Chongqing, Guizhou, Yunnan, Guangxi Zhuang Autonomous Region, Shaanxi, and Gansu. Food consumption was recorded using a discontinuous 3-day, 24 h method, face-to-face interviews of 24 h diet recalls that were performed on one weekend day (Saturday or Sunday) and two weekdays, and two surveys were conducted at least three days apart. A total of 55,700 individuals participated in the survey, yielding 58,610 person-days of data for seafood and fermented food consumption. The age range of seafood and fermented food consumers was 3 to 92 years.

#### 2.6.2. Acute Dietary Exposure to Five BAs from Seafood and Fermented Foods Among Chinese Consumers

In this study, a point assessment model was employed to estimate the acute dietary exposure to five BAs from seafood and fermented foods in China. Acute exposure to these BAs under the worst-case consumption scenario was calculated based on the P99 of daily consumption of seafood and fermented foods and the P95 of the concentrations of the five BAs in these foods. Furthermore, based on the WHO-recommended approach for undetected values, the worst-case scenario was used to conservatively estimate acute dietary exposure to the five BAs by consumers; the undetected values were assumed to be at LOD [26].

The acute dietary exposure to BAs were assessed according to the following formula:

(1) $$\;EXP=\frac{{Com}_{i}\times {Con}_{k}}{1000}$$

where *EXP* denotes the acute dietary exposure to the five BAs from seafood and fermented foods in consumers (mg/d); ${Com}_{i}$ denotes the P99 of the daily consumption of the seafood and fermented foods (g/day); ${Con}_{k}$ denotes the P95 of the concentrations of the five BAs in seafood and fermented foods (mg/kg).

In this study, the acute dietary exposure to HIS from seafood and fermented foods was compared with the temporary ARfD of HIS established by EFSA (50 mg/d) [10]. Consumers with an acute dietary exposure to HIS of more than 50 mg/d indicated a possible acute health risk.

#### 2.6.3. Probabilistic Estimation of Acute Dietary Exposure to HIS Among Cheese and Fermented Bean Product Consumers in China

Acute dietary exposure to HIS from cheese and fermented bean products consumed by Chinese consumers was calculated probabilistically using R software (version 4.4.0). The probabilistic modeling used in this study was implemented via Monte Carlo simulations, which simulated daily consumption by sampling from the consumption distributions of cheese and fermented bean products and combining these data with random samples from the distribution of HIS concentrations. Random sampling from the concentration distribution was performed based on the percentage of samples with detectable concentrations and the percentage of samples with undetectable concentrations. Each consumer’s acute dietary exposure (expressed as mg/d) was calculated by combining the consumptions of fermented bean products and cheese with the corresponding concentrations of HIS.

R software was employed to fit the HIS concentration data, as well as the consumption data for fermented bean products and cheese, to appropriate distributions. A lognormal distribution was used to fit the HIS concentrations above the limit of detection (LOD). Considering the uncertainty and variability of the HIS concentrations in undetected samples, HIS concentrations below the LOD were fitted using a uniform distribution ranging from 0 to the LOD [27,28]. The consumption data of cheese and fermented bean products were fitted using a lognormal distribution.

Probabilistic modeling was implemented using Monte Carlo simulations, which simulate daily consumption by sampling from the consumption distributions of cheese and fermented bean products and combining these data with random samples from the distribution of HIS concentrations. Each consumer’s acute dietary exposure to HIS (expressed as mg/d) was calculated by combining the consumption amounts of fermented bean products and cheese with the corresponding HIS concentrations. Detailed information on the variables and models used for this acute exposure assessment is shown in Table 3. The number of Monte Carlo iterations was set to 100,000, and the bootstrap sample was 100. The acute dietary exposures were specified at the percentiles P25, P50, P75, P90, P95, P97.5, P99, and P99.9, as well as the average value from the intake distribution. These exposure estimates were also compared with the temporary ARfD of 50 mg/d HIS for a healthy individual, as established by EFSA.

### 2.7. Statistical Analysis

Data analysis was conducted using SPSS (version 25.0 for Windows, Armonk, NY: IBM Corp., USA). The concentrations of BAs in the samples are presented as the mean and P95 values of the detected BAs. Acute dietary exposure assessments for HIS among Chinese consumers were conducted using R software. Additionally, multivariate data analysis was conducted using R (Version 4.4.0) to investigate potential patterns in the concentrations of five BAs in high-HIS marine fish and general marine fish, including their correlation structures. Correlations with *p* < 0.05 were considered statistically significant.

## 3. Results and Discussion

### 3.1. Concentrations of Five BAs in Seafood, Fermented Foods, and Complementary Foods for Infants and Young Children

A total of 1200 samples of seafood, fermented food, and complementary food for infants and young children collected in China were analyzed. Table 4 presents the concentrations of the five BAs in these food samples, including the detection rates and the concentrations. HIS exhibited the highest detection rate at 51.9%, followed by PUT at 50.1%, CAD at 46.3%, TYR at 44.1%, and TRY at 12.5%. The detection rate of HIS in Huangjiu and shrimp paste was more than 90% in both. The top three average concentrations of HIS were found in fish sauce, other fermented bean products, and fermented bean curd, especially in fish sauce samples, where the P95 concentration of HIS reached 903.5 mg/kg. Among the other fermented bean product samples, the highest concentration of HIS was found in the moldy tofu sample (633 mg/kg), while the concentrations of HIS in stinky tofu and soybean paste were low. For HIS, which is the most toxic of the BAs, our study suggested that fish sauce, moldy tofu, and fermented bean curd are fermented foods that require more attention. In the EFSA’s report, the results indicated that dried anchovies, fish sauce, and fermented vegetables were foods with higher concentrations of HIS [10]. Both our and EFSA’s studies show that the concentration of HIS in fish sauce samples was higher than that in other fermented foods [10]. This might be due to factors such as the raw materials, production process, and bacterial communities used in the production of fish sauce [29]. In addition, fermented bean curd and moldy tofu, as traditional Chinese fermented bean products, contain BAs, especially HIS, which have become potential risk factors. Several studies have indicated that the abundant amino acid components in fermented bean products, together with their specific fermentation methods, can result in higher levels of BA contamination in certain products [30,31]. Thus, researchers need to develop innovative techniques to control BA levels in fermented bean products.

**Figure 1:**
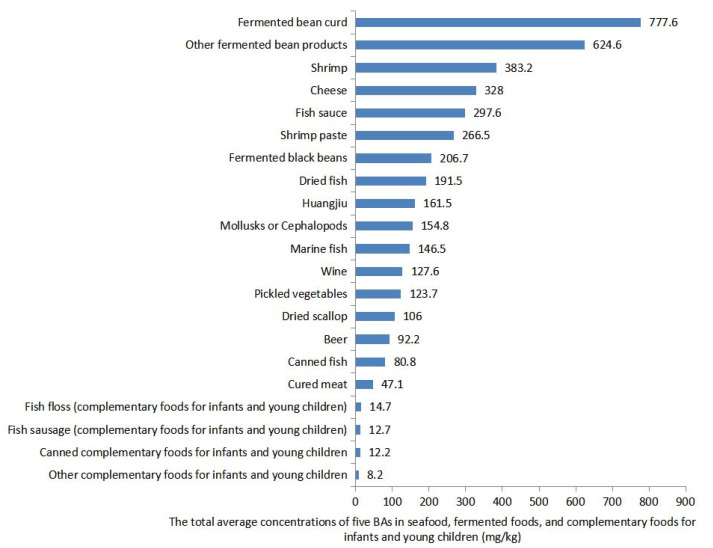
The total average concentrations of five BAs in seafood, fermented food, and complementary food for infants and young children.

For the other four types of BAs, the detection rate of TYR in other fermented bean product samples was 100%, and both the average and P95 concentrations of TYR were highest in the fermented bean curd samples. In addition to HIS, TYR in fermented bean curd also needs attention. Additionally, the analysis of the total average concentration of the five BAs in the samples revealed that fermented bean curd had the highest total BA concentration at 777.6 mg/kg, followed by shrimp and cheese (Figure 1). In contrast, the concentrations of the five BAs and total average concentration of the five BAs in complementary foods for infants and young children were lower than in other samples. For PUT, CAD, and TRY, fermented bean curd, cheese, and dried scallops were the foods with the highest average concentrations, respectively. Although PUT, CAD, and TRY have lower toxicity, the above-mentioned foods may need more attention for sensitive consumers.

### 3.2. Concentrations of Five BAs in High-HIS Marine Fish and General Marine Fish

**Figure 2:**
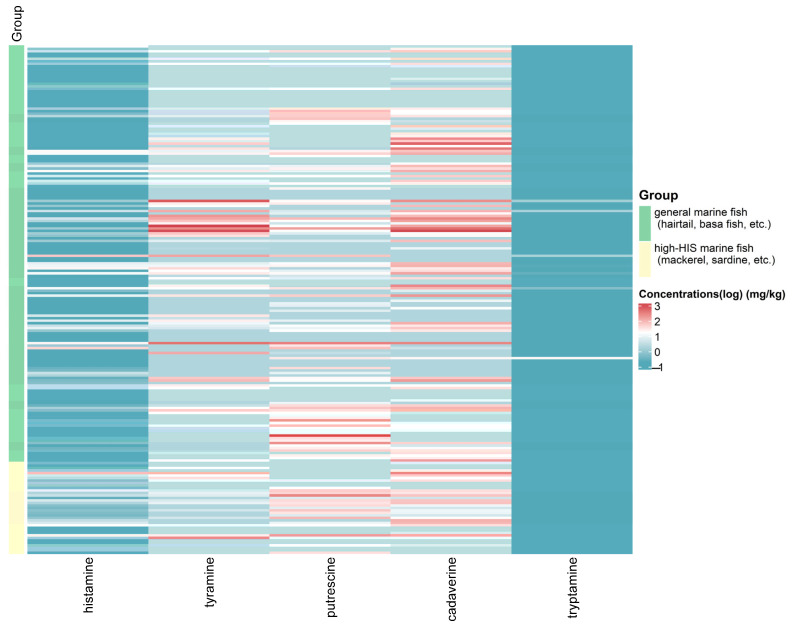
Heatmaps of log-concentrations of five BAs in high-HIS marine fish and general marine fish.

**Figure 3:**
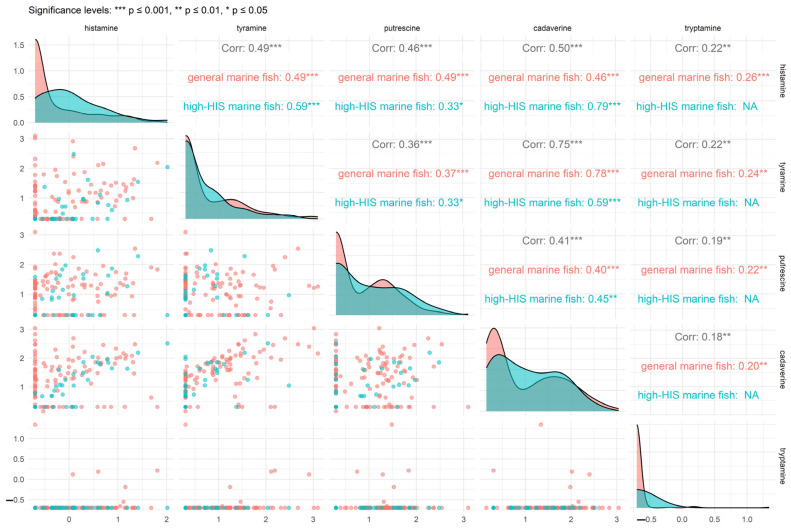
Data analysis on log-concentrations of BAs in high-HIS marine fish and general marine fish.

In order to analyze the differences in the contamination spectrum of BAs between high-HIS fish and general marine fish, as well as the correlations between the five BAs, we conducted a multivariate analysis of the concentrations of BAs in both types of marine fish. Figure 2 revealed distinct patterns in the concentrations of the five BAs between high-HIS marine fish and general marine fish. In this study, TYR, PUT, and CAD had higher concentrations in the two types of marine fish samples, while HIS and TRY had lower concentrations. There was no significant difference in concentration of HIS between the high-HIS marine fish and general marine fish. The reason might be that the freshness and storage conditions of the two types of marine fish samples were similar [32]. To further investigate these heterogeneous patterns and the potential correlation structure of HIS and other BAs, a multivariate data analysis was conducted. As shown in Figure 3, a pairs plot was employed to analyze the internal structure of the sample data [33]. The estimated density functions in the diagonal blocks indicated that HIS and TRY displayed approximately right-skewed distributions, whereas the other three BAs showed bimodal distributions in both types of marine fish. The concentration distribution of TYR was similar between the two types of marine fish. Differences in amino acid ratios and microbial communities in marine fish result in varying concentration distributions of BAs.

Additionally, the Spearman correlation test indicated significant correlations among the five BAs, with the strongest correlation observed between CAD and TYR (0.749), followed by HIS and CAD (0.499), and HIS and TYR (0.485). In this study, the strong correlation between CAD and TYR in marine fish may be related to specific microorganisms, appropriate storage conditions, and the similar enzymatic generation mechanism [34]. To better summarize and compare the variations in the correlation structures of the five BAs across the two types of marine fish, Figure S2 presents heatmaps of the correlation matrix for each type. As shown in Figure S2, the correlation structures of the five BAs differed between high-HIS marine fish and general marine fish. In high-HIS marine fish, the strongest correlation was observed between HIS and CAD, whereas in general marine fish, the highest correlation was between TYR and CAD. In contrast, TRY exhibited generally low correlations with the other BAs, with some correlations failing to reach statistical significance.

### 3.3. Acute Dietary Exposure to Five BAs from Seafood and Fermented Food Among Chinese Consumers

The results of the point assessment are presented in Table 5. According to Table 5, acute dietary exposure to HIS was highest from cheese (76.2 mg/d), followed by fermented bean products (74.5 mg/d), both of which exceeded the ARfD of HIS established by EFSA (50 mg/d). Acute dietary exposure to TYR was highest from seafood (184.0 mg/d), followed by fermented bean products (146.7 mg/d) and cheese (141.2 mg/d). Based on the limited clinical trial data available, EFSA has suggested that healthy adults who are not taking monoamine oxidase inhibitor (MAOI) drugs are unlikely to experience adverse health effects when consuming up to 600 mg of TYR per person per meal. For healthy Chinese adults, even in the worst-case scenario, the risk of acute TYR poisoning remains relatively low. Acute dietary exposure to PUT was highest from cheese (482.1 mg/d), followed by fermented alcoholic drinks (361.3 mg/d) and processed seafood products (166.7 mg/d). Acute dietary exposure to CAD was highest from fermented bean products (144.0 mg/d), followed by processed seafood products (122.6 mg/d) and seafood (104.3 mg/d). Acute dietary exposure to TRY was highest from processed seafood products (58.9 mg/d), followed by fermented bean products (38.6 mg/d) and seafood (10.1 mg/d). Therefore, consumers with high intake of cheese or fermented bean products (e.g., fermented black beans and fermented bean curd) may be at an increased risk of HIS intoxication. However, the acute exposure assessment of HIS was conducted using a point assessment method, which represents a worst-case scenario. To minimize uncertainties and provide a more accurate assessment, a probabilistic assessment method was employed to evaluate acute HIS exposure from cheese and fermented bean products.

A comparison of the results from this study with EFSA’s 2011 [10] report showed some differences. EFSA also employed a point assessment method—using the P95 of food consumption and the P95 of HIS concentration to estimate high acute dietary exposure to HIS. For European consumers, the top three foods contributing to high acute dietary exposure to HIS were other fish and fish products (including fresh, frozen, or canned fish meat that did not undergo a fermentation process), fermented sausages, and cheese [10]. The differences in dietary habits between Chinese and European consumers may be one reason for this. Chinese consumers prefer fermented bean products in their diet, while European consumers tend to prefer seafood and dairy products. Furthermore, the acute dietary exposures to PUT and CAD in cheese and fermented bean products are higher than those in other foods. Due to the lack of a dose–response relationship between the concentrations of PUT and CAD in foods and their acute health effects in humans, this study is unable to estimate the acute health risks associated with PUT and CAD in foods [10]. However, considering that PUT and CAD potentiate the toxicity of other amines, especially HIS, it is necessary to control both of them in cheese and fermented bean products.

### 3.4. Probabilistic Estimation of Acute Dietary Exposure to HIS from Cheese and Fermented Bean Products Among Chinese Consumers

Acute dietary exposure to HIS from cheese and fermented bean products among Chinese consumers was further analyzed using probabilistic assessment methods. The results of the probabilistic assessment are presented in Table 6. For consumers of fermented bean products, the average and high (P99) acute dietary exposures to HIS were 1.57 mg/d and 24.98 mg/d, respectively. Among cheese consumers, the average and high (P99) acute dietary exposures to HIS from cheese were 0.12 mg/d and 1.92 mg/d, respectively. Additionally, based on the ARfD of HIS (50 mg/d) [10], the probability of acute health risks from dietary HIS exposure was estimated to be 0.44% for fermented bean product consumers and 0.014% for cheese consumers, respectively. These findings indicate that while the overall acute health risk of HIS exposure from seafood and fermented foods is low for the general Chinese population, individuals with high consumption of cheese and fermented bean products may need to be concerned. Meanwhile, based on the ARfD of HIS (50 mg/d) and P99 consumption of cheese and fermented bean products (120 g for both cheese and fermented bean products), the levels of concern (LOC) for HIS in cheese and fermented bean products were calculated to be 416.7 mg/kg, respectively. If the concentration of HIS in foods exceeds the LOC, it indicates that there may be a health risk for consumers. In this study, the concentration of HIS in one sample of cheese and four samples of fermented bean products exceeded the LOC (416.7 mg/kg). Among the fermented bean product samples, the concentrations of HIS in both fermented bean curd and moldy tofu samples exceeded the LOC for HIS, indicating that these fermented bean products may require more attention.

### 3.5. Uncertainty Analysis

Some uncertainties in this study may influence the estimated exposure to HIS. Firstly, the types of food monitored did not fully cover all types of foods that may contain the five BAs, such as soy sauce and other seafood products. Additionally, the proportion of consumers of some foods was low, and the under-representation of corresponding food consumption causes uncertainties in the resulting risk. Secondly, in the probabilistic assessment, samples with HIS levels below the LOD were assumed to be at the LOD, which may potentially overestimate HIS exposure from these foods. Thirdly, there is limited information on the dose–response relationship for HIS causing adverse health effects [10]. For instance, experimental results in healthy volunteers and sensitive individuals have shown variability, with differences in sensitivity both within and between individuals, which affects the reproducibility of the experimental results. Additionally, the synergistic toxicity of PUT and CAD with HIS in foods may bring uncertainty to the acute health risk assessment of HIS. Finally, due to the limitations of the LC-MC analysis used in this study, only the five major BAs in the main foods were analyzed, while other BAs, such as phenethylamine, spermidine, and spermidine were not included. This selective analysis may therefore not fully represent the overall BA exposure profile.

## 4. Conclusions

The current study determined the concentrations of five BAs in seafood, fermented foods, and complementary foods for infants and young children from Chinese markets using the HPLC-MS-MS method and estimated the acute health risks of HIS from fermented foods to Chinese consumers. Among all the samples, HIS had the highest detection rate, followed by PUT, CAD, TYR, and TRY. The analysis of the total average concentrations of the five BAs in seafood, fermented food, and complementary food for infants and young children indicated that fermented bean curd had the highest total BA content, followed by shrimp and cheese. In contrast, complementary foods for infants and young children had the lowest levels. In the point assessment, acute dietary exposure to HIS from cheese and fermented bean products exceeded the ARfD established by EFSA. For Chinese consumers, the probability of acute health risks from exposure to HIS was 0.44% for fermented bean product consumers and 0.014% for cheese consumers, respectively. In addition, individuals with high consumption of cheese or fermented bean products may need to be concerned. Therefore, considering that HIS in fermented bean products may pose acute health risks to consumers, it is essential to control the fermentation process and monitor BAs during the production and storage of fermented bean products.

## Figures and Tables

**Table 1:** Gradient elution program.

Time (min)	A (%)	B (%)
0.0	0	100
2.0	0	100
4.5	55	45
5.5	55	45
5.6	0	100
9.0	0	100

**Table 2:** Retention time, qualitative ions, quantitative ions, and collision energies of five BAs.

BAs	Retention Time (min)	Qualitative Ion (*m*/*z*)	Quantitative Ion (*m*/*z*)	Collision Energy (eV)
CAD	5.49	103.0 > 86.1 103.0 > 69.0	103.0 > 86.1	15 22
PUT	5.49	89.1 > 72.0 89.1 > 30.0	89.1 > 72.0	13 31
TYR	3.29	138.2 > 121.1 138.2 > 77.0	138.2 > 121.1	16 34
HIS	5.46	112.2 > 95.1 112.2 > 68.0	112.2 > 95.1	20 30
TRY	2.94	161.2 > 144.1 161.2 > 117.0	161.2 > 144.1	18 33

**Table 3:** Description and distribution of variables and models for acute dietary exposure assessment of HIS from cheese and fermented bean products in Chinese consumers.

Variables	Definition	Assumption/Distribution/Formula	Source
*Con_ds_*	Concentrations of HIS in detected samples	Lognorm (2.52, 2.01) ^a^ Lognorm (0.84, 1.96) ^b^	Monitoring data
*Con_us_*	Assumed concentrations of HIS in undetected samples	Uniform (0, 0.2)	Monitoring data
*P_p_*	Rate of samples with detectable concentrations	78.77% ^a^ 27.16% ^b^	Calculated
*P_n_*	Rate of samples with undetectable concentrations	21.23% ^a^ 72.84% ^b^	Calculated
*Con*	Concentrations of HIS in samples	Discrete (*Con_us_*:*Con_ds_*, *P_n_*:*P_p_*)	Calculated
*Com*	Consumption	Lognorm (2.62, 0.92) ^a^ Lognorm (3.02, 0.62) ^b^	China National Food Consumption survey (2018–2020)

Note: ^a^: fermented bean products; ^b^: cheese.

**Table 4:** Concentrations of five BAs in seafoods, fermented foods, and complementary foods for infants and young children.

Food Category		HIS			TYR			CAD			PUT			TRY		
		Detection Rate (%)	Concentration (mg/kg)		Detection Rate (%)	Concentration (mg/kg)		Detection Rate (%)	Concentration (mg/kg)		Detection Rate (%)	Concentration (mg/kg)		Detection Rate (%)	Concentration (mg/kg)	
			Mean	P95		Mean	P95		Mean	P95		Mean	P95		Mean	P95
Seafood	Marine fish	48	5.9	22.6	43.1	72.8	400.1	54.9	84.7	314.7	50.0	52.3	226.7	3.4	3.9	\
	Shrimp	26.2	1.4	10.1	36.0	164.8	847.5	42.4	226.0	1092.0	39.0	171.9	1153.4	8.7	1.5	\
	Mollusks or Cephalopods	19.3	8.1	46.3	21.1	62.6	287.8	35.8	95.3	657.0	42.2	94.1	546.7	9.0	10.4	\
Processed seafood products	Dried fish	68.6	12.2	120.7	51.4	23.7	\	74.3	106.9	555.6	57.1	92.8	408.7	14.3	1.1	\
	Dried scallops	34.6	3.1	\	7.7	79.9	\	34.6	35.3		46.2	47.5	\	3.8	196.4	\
	Shrimp paste	93.8	17.8	\	75.0	119	\	68.8	123.2		75.0	73.2	\	56.3	37.3	\
	Canned fish	85.7	8.8	57.2	62.9	30.1	93.2	62.9	38.3	214.4	60.0	38.6	125.3	17.1	1.1	\
Condiments	Fish sauce	71.1	118.0	903.5	39.5	44.4	\	55.3	129.5	497.8	44.7	93.5	\	36.8	12.4	\
Complementary foods for infants and young children	Fish sausage	88.5	2.7	22.2	30.8	3.7	\	50.0	14.6	\	11.5	7.9	\	\	\	\
	Fish floss	80.8	5.8	20.6	38.5	4.8	\	23.1	13.3	\	26.9	10.3	\	7.7	0.4	\
	Canned complementary foods for infants and young children	36.4	0.6	\	9.1	2.5	\	4.5	5.9	\	90.9	11.9	39.3	13.6	0.5	\
	Other complementary foods for infants and young children	81.8	0.5	\	81.8	2.8	\	36.4	6.8	\	9.1	25.5	\	0	\	\
Fermented milk products	Cheese	27.2	38.7	634.9	27.2	255.2	1176.8	16.0	576.2	\	40.7	64.0	771.3	6.2	1.4	\
Fermented alcoholic drink	Huangjiu	96.9	8.3	34.9	87.5	33.9	86.0	53.1	26.7	\	100.0	109.6	401.41	0	\	\
	Beer	29.4	0.3	\	44.1	3.1	\	41.2	20.3	\	52.9	88.2	\	2.9	14.5	\
	Wine	33.3	10.7	\	30.0	3.4	\	23.3	6.0	\	33.3	122.4	\	0	\	\
Fermented vegetable products	Pickled vegetables	76.3	23.6	90.6	61.2	44.6	161.0	48.7	81.6	439.8	63.8	42.8	199.9	18.4	4.0	23.1
Fermented meat products	Cured meat	59.2	6.4	29.3	44.7	21.9	79.5	55.3	37.6	154.8	38.2	12.9	45.1	11.8	3.2	\
Fermented bean products	Fermented black beans	72.0	26.0	161.5	65.6	82.4	479.6	50.0	154.7	\	56.3	56.2	\	34.4	35.0	\
	Fermented bean curd	86.8	94.8	598.8	81.6	298.4	1198.0	81.6	172.0	921.5	84.2	322.3	1226.0	52.6	76.3	262.4
	Other fermented bean products	80.0	108.2	430.8	100	240.6	869.0	70.0	108.2	430.8	60.0	93.9	198.8	50.0	112.1	346.8
Total		51.9	22.0	80.4	44.1	87.1	478.5	46.3	114.4	522.4	50.1	86.6	368.4	12.5	23.3	145.5

Note: Canned complementary foods refer to canned complementary foods for infants and young children containing fish and shrimp ingredients; other complementary foods for infants and young children include shrimp patties, shrimp slices, fish puff balls; other fermented bean products include stinky tofu, moldy tofu, and soybean paste. Detection rate (%) = (Number of positive samples/Total number of samples of each type of food) × 100%; P95 means 95th percentile of HIS concentration in food samples; symbol “\” indicates that due to limited sample size, 95th percentile could not be reliably estimated and is therefore not reported.

**Table 5:** Estimation of acute dietary exposure to five BAs from seafood and fermented foods among Chinese consumers.

Food Category	Consumption (g/d)	HIS		TYR		PUT		CAD		TRY	
		Concentration (mg/kg)	Exposure (mg/d)	Concentration (mg/kg)	Exposure (mg/d)	Concentration (mg/kg)	Exposure (mg/d)	Concentration (mg/kg)	Exposure (mg/d)	Concentration (mg/kg)	Exposure (mg/d)
Seafoods	460	22.6	10.4	400.1	184.0	314.7	144.8	226.7	104.3	21.9 *	10.1
Processed seafood products	300	120.7	36.2	319.2 *	95.8	555.6	166.7	408.7	122.6	196.4 *	58.9
Cheese	120	634.9	76.2	1176.8	141.2	4017.8 *	482.1	771.3	92.6	4.5	0.5
Fermented alcoholic drink	900	34.9	31.41	86	77.4	401.41	361.3	98.9 *	89.01	0	0
Fermented vegetable products	204	90.6	18.5	161.0	32.8	439.8	89.7	199.9	40.8	23.1	4.7
Fermented meat products	300	29.3	8.8	79.5	23.9	154.8	46.4	45.1	13.5	23.9	7.2
Fermented bean products	120	621	74.5	1222.7	146.7	1356	162.7	1199.8	144.0	321.4	38.6
Fish sauce	5	903.5	4.5	112.3 *	0.6	497.8	2.5	294 *	1.5	87.2	0.4

Note: The point assessment of acute dietary exposure was calculated using the P99 of consumption data and P95 of BA concentrations; values marked with an asterisk (*) represent the maximum concentrations of the five BAs (some samples lacked P95 values for BA concentrations).

**Table 6:** Probabilistic estimation of acute dietary exposure to HIS from cheese and fermented bean products among Chinese consumers.

Foods	Acute Dietary Exposure to HIS (mg/d)									95% CI
	Mean	P25	P50	P75	P90	P95	P97.5	P99	P99.9	
Fermented bean products	1.57	0.01	0.08	0.49	2.11	5.06	10.60	24.98	135.59	10.37–10.46
Cheese	0.12	0.001	0.003	0.01	0.98	0.31	0.75	1.92	11.47	0.72–0.73

Note: 95% CI, confidence interval of P97.5. Fermented bean products include fermented black beans, fermented bean curd, and other fermented bean products (such as stinky tofu, moldy tofu, and soybean paste). Cheese includes goat’s milk cheese and cow’s milk cheese.

## Data Availability

The original contributions presented in the study are included in the article/supplementary material, further inquiries can be directed to the corresponding author.

## Supplementary Materials

The following supporting information can be downloaded at https://www.mdpi.com/article/10.3390/foods14142550/s1, Figure S1: Quantitative ion chromatograms of standard solutions of cadaverine (CAD), putrescine (PUT), tyramine (TYR), histamine (HIS), and tryptamine (TRY); Figure S2: Heatmaps of correlation matrix of BAs for all samples in different marine fish groups.
