# Supplementary material for: Analysis of Five Biogenic Amines in Foods on the Chinese Market and Estimation of Acute Histamine Exposure from Fermented Foods in the Chinese Population
Source: Foods. 2025 Jul 21;14(14):2550. doi: 10.3390/foods14142550 (PMC12294725; doi:10.3390/foods14142550)
Supplement: Supplementary file 1 [file foods-14-02550-s001.zip › foods-3700433-supplementary.pdf]

Supplementary document

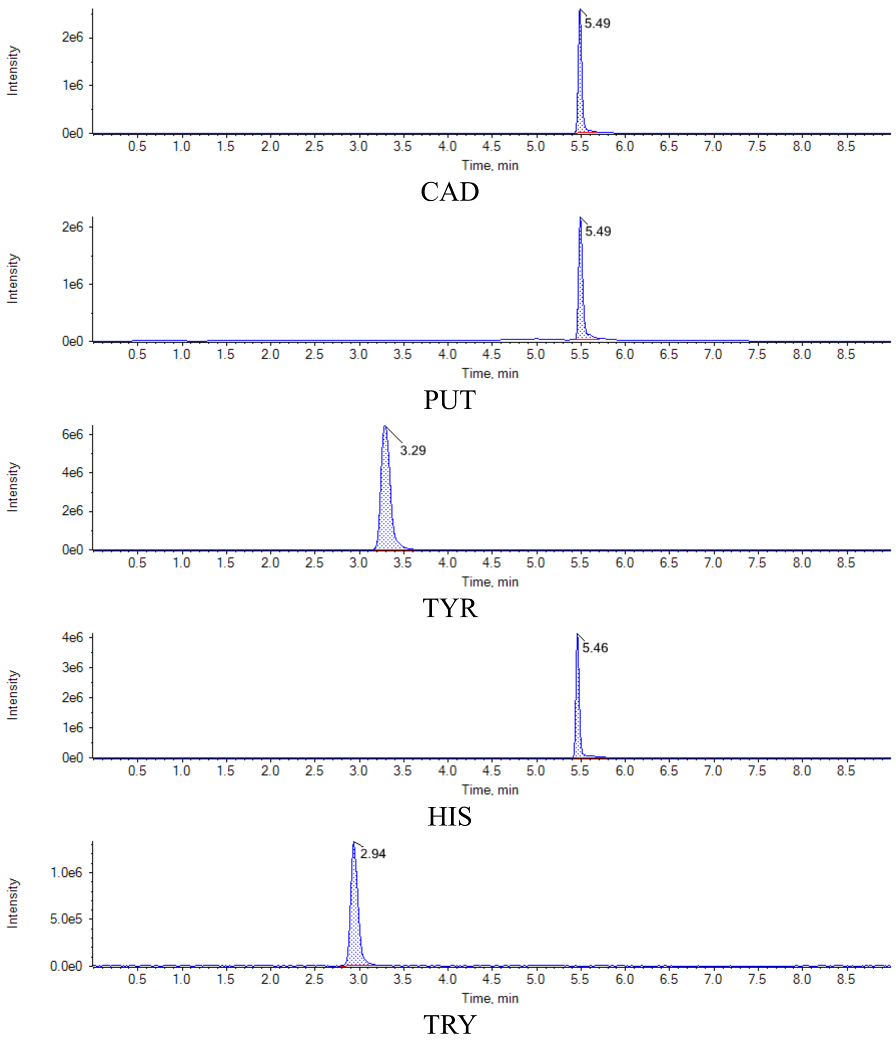

Figure S1. Quantitative ion chromatograms of standard solutions of cadaverine (CAD), putrescine (PUT), tyramine (TYR), histamine (HIS), and tryptamine (TRY) (CAD, TYR, and PUT at 500 μg/L; HIS and TRY at 50 μg/L).

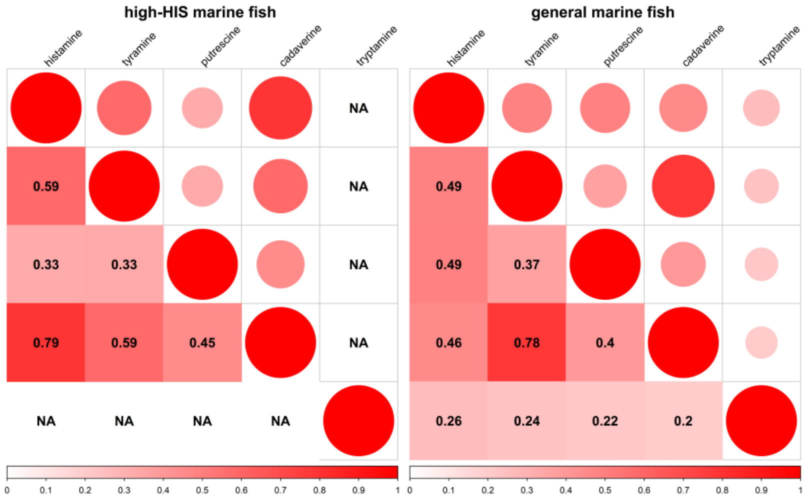

Figure S2. Heatmaps of correlation matrix of BAs for all samples in different marine fish groups. The strength of the correlation is indicated by both the color intensity and the size of the circles, with deeper red and larger circles representing stronger positive correlations. Correlation coefficients (R^2) were calculated using Spearman's rank correlation. "NA" indicates that there are too many missing data to calculate the correlation coefficients in the according cells.
